# Characterization of the Culturable Subpopulations of *Lactobacillus* in the Chicken Intestinal Tract as a Resource for Probiotic Development

**DOI:** 10.3389/fmicb.2017.01389

**Published:** 2017-07-26

**Authors:** Bishnu Adhikari, Young M. Kwon

**Affiliations:** ^1^Department of Poultry Science, College of Agricultural, Food and Life Sciences, University of Arkansas, Fayetteville AR, United States; ^2^Cell and Molecular Biology Program, University of Arkansas, Fayetteville AR, United States

**Keywords:** broiler, gastrointestinal tract, *Lactobacillus*, microbiota, probiotics

## Abstract

To gain better understanding of the distributions of the culturable *Lactobacillus* species in the chicken intestinal tract, we collected ceca, and distal ileum from 10 3-weeks-old broiler chickens. *Lactobacillus* strains from cecal lumen contents (M-CL), and those associated with mucosa of ceca (M-CM) and ileum (M-IM) were recovered on de Man, Rogosa and Sharpe (MRS) agar plates, and used for microbiota analysis. The total cecal content (T-CL) was also used directly for microbiota analysis. We purposefully focused on MRS-recovered populations to gain understanding of the culturable subpopulations of *Lactobacillus*, since the culturability is an important phenotype in order to exploit the chicken gut microbiota as a resource for development of probiotics. The V1–V3 regions of 16S rRNA gene was amplified from genomic DNA samples, and the pooled amplicons were analyzed by MiSeq sequencing with paired-end read 300 cycle option. Among MRS groups, *Firmicutes* were significantly higher in M-IM and M-CL as compared to M-CM, whereas *Proteobacteria* were significantly higher in M-CM as compared to M-IM and M-CL at *p* < 0.05. Among *Lactobacillus*, *L. salivarius* (36%) and *L. johnsonii* (21%) were higher in M-IM as compared to M-CL (*L. salivarius*, 28%; *L. johnsonii*, 15%), and M-CM (*L. salivarius*, 20%; *L. johnsonii*, 11%). *L. crispatus* was found significantly higher in M-CL as compared to M-IM (*p* < 0.01) whereas *L. gasseri* was found significantly higher in M-IM as compared to M-CM (*p* < 0.05). *L. aviarius*, and *L. fornicalis* were only observed in T-CL. In summary, *Lactobacillus* populations recovered on MRS vary with different regions and locations in chicken GIT, which might indicate their distinct functional roles in different gastrointestinal tract (GIT) niches, and some species of *Lactobacillus* are not culturable on MRS agar media. This study is the first attempt to define culturable *Lactobacillus* subpopulations in the chicken intestinal tract comprehensively using 16S rRNA gene profiling, and the findings of this study will be used as a platform to develop a new strategy for isolation of effective *Lactobacillus* probiotic candidates based on comparative analyses of chicken gut microbiota.

## Introduction

Due to the increased risk associated with the development of antibiotic resistance in bacteria, the use of antibiotic growth promoters (AGPs) in animal industry has been completely banned in Europe since January 1, 2006 and has been in the process of reduction or complete elimination in several countries, including the United States (Dibner and Richards, 2005; Huyghebaert et al., 2011). The use of probiotics as an alternative to AGP has been rapidly increasing in recent years (Ahasan et al., 2015). Microbes that are commonly used as probiotics include various species of the genera *Lactobacillus*, *Bifidobacterium*, and *Enterococcus* (Moreira et al., 2005). Although the microbial communities are distributed throughout the GIT, their composition was found heterogeneous along the different regions of GIT in chicken (Yeoman et al., 2012; Choi et al., 2014; Ranjitkar et al., 2016), pigs (Looft et al., 2014), and cattle (Mao et al., 2015). The variations in microbial composition can occur not only in different segments along GIT, but can also at different locations (lumen vs. mucosa) in the same region (Gong et al., 2002; Looft et al., 2014). Diverse groups of microbes reside in various regions and locations of the GIT and this might indicate differential functional roles they play in maintaining host health. Thus, in this study we characterized the bacterial communities across the different regions and locations of the GIT of chickens with a focus on the genus *Lactobacillus*, which have been most commonly considered for probiotics, through microbiota analysis of the bacterial cells recovered on MRS agar plates. By characterizing bacterial cells recovered on MRS agar plates, we eliminate unculturable *Lactobacillus* strains from the downstream analysis, retaining only culturable strains. If necessary, this step can be followed by identification and isolation of the species that demonstrate promising utility as probiotics based on comparative metagenomic analysis (16S rRNA gene profiling, and/or shot-gun metagenomics). For example, when a comparative microbiota/microbiome analysis indicates particular species (or strains) as effector species (or strains), the culturability of the corresponding species can be first confirmed by the presence of corresponding DNA signatures in culture-recovered bacterial populations before any attempt can be made to isolate the target species (strains) for further evaluation as promising probiotics. It is important to note that current method for 16S rRNA gene profiling using Illumina sequencing has a limited resolution and often cannot differentiate even at a species-level, while a strain-level analysis is impossible. It is mainly due to short lengths of the target regions in 16S rRNA gene that are sequenced, and inevitable sequencing errors from PCR and sequencing step. However, with the increasing interest in exploring intra-species variations, novel methods have been developed to overcome the current limitations enabling microbiota analysis at a strain-level (Ellegaard and Engel, 2016).

*Lactobacillus* strains were found to enhance tight junctions, and thereby reducing intestinal permeability in both *in vitro* studies with Caco-2 cells (Anderson et al., 2010; Miyauchi et al., 2012) and *in vivo* study with mice (Xu et al., 2016). However their distribution at species level, and functional activity may differ in different regions and locations of the GIT. *Lactobacillus* strains that are tightly associated with mucosa might possess better properties as probiotics than those found in lumen, and detailed characterization of *Lactobacillus* populations in both lumen and mucosa of different regions may be very helpful in the quest for isolating good probiotic candidates. Although MRS agar is the most commonly used medium for isolation of *Lactobacillus* strains, the scope of the culturability on MRS agar for diverse *Lactobacillus* species has not been systematically evaluated. In addition, since the use of candidate *Lactobacillus* strains for probiotic applications would require the culturability of the strains, in this study we adopted the approach of characterizing *Lactobacillus* strains recovered on MRS agar plates.

The precise identification of *Lactobacillus* isolates by phenotypic method is difficult, because phenotypic properties beyond the common fermentation tests are often required, and around 17 phenotypic tests are required to identify *Lactobacillus* at species level (Moreira et al., 2005). Only around 30% of the total vaginal and intestinal lactobacilli from humans were identified correctly at the species level by the most commonly used commercially available biochemical kit (Song et al., 1999). Alternatively, taxonomic identification of the strains belonging to genus *Lactobacillus* can be performed at species level with high accuracy based on DNA sequencing of the variable regions in 16S ribosomal RNA (16S rRNA) gene (Woo et al., 2002; Piotrowska et al., 2016).

Hence, the main aim of this study is to analyze bacterial populations recovered on MRS agar media via deep sequencing of the V1–V3 region of 16S rRNA gene in order to better understand the structure and distribution of the culturable subpopulations of *Lactobacillus* in different regions and locations of the GIT of broiler chickens.

## Materials and Methods

### Sample Collection and Processing

Cobb 500 broiler chickens were provided *ad libitum* access to water and an antibiotic-free corn-soybean meal diet. At the age of 3 weeks, 10 birds were humanely sacrificed, and ceca and distal end of ileum (5 cm) were aseptically collected according to the animal use protocol (No. 16047) approved by the IACUC committee at the University of Arkansas. The age of 3 weeks was chosen because the gut microbiota are established stably around this age (Ranjitkar et al., 2016). Cecal lumen contents were serially diluted and plated on MRS agar plates. To isolate bacteria associated with cecal mucosa or ileal mucosa, each mucosa sample was washed in sterile PBS buffer (pH 7.4) after removing luminal contents for four times, and homogenized in 20 ml PBS using Bullet Blender^®^ (Next Advance). The supernatant was collected, serially diluted, and plated on MRS agar plates. The MRS agar plates were incubated overnight at 37°C under microaerophilic condition. Bacterial pellets were recovered from MRS plates with lowest dilutions (1 plate per sample) by resuspending all colonies in 5 ml PBS followed by centrifugation. The lowest dilutions were used to maximize the number of colonies collected for each sample: 10-fold dilution was used for M-CL and the supernatant without dilution was for M-CM and M-IM. The average log_10_CFUs per sample (mean ± standard error) was 6.02 ± 0.18, 3.71 ± 0.18, and 3.23 ± 0.21 for M-CL, M-CM, and M-IM samples, respectively.

### DNA Extraction and PCR

Genomic DNA was extracted from each pellet (equal amount) by using DNeasy Blood and Tissue Kit (Qiagen). Genomic DNA of total bacteria in cecal lumen was also extracted directly without culturing on MRS plates using QIAamp Fast DNA Stool Minikit (Qiagen). Thus, we had altogether 40 genomic DNA samples: 10 MRS-recovered cells from each of cecal lumen (M-CL), cecal mucosa (M-CM), and ileal mucosa (M-IM), and 10 total bacterial cells from cecal lumen (T-CL). The V1–V3 region of the 16S rRNA gene was amplified from the genomic DNA samples using barcode-tagged universal primers; 27F (5′-AGRGTTYGATYMTGGCTCAG-3′) and 533R (5′-TTACCGCGGCTGCTGGCAC-3′) with attached Illumina adapters. Details regarding primers, enzymes, and PCR conditions were previously described (Mandal et al., 2016). The amplicons were purified from 0.7% agarose gel electrophoresis after verifying the length of amplicons. After the concentration of each amplicon sample was measured using Qubit dsDNA broad range assay kit (Life Technologies, United States), the amplicons were pooled at an equal amount. The pooled sample was gel-purified from 6% TBE gel (Invitrogen, United States), and sent for Illumina sequencing at the University of California (Riverside, CA, United States) using MiSeq paired-end reads with 300 cycles.

### Data Analysis

All MiSeq paired-end sequence reads were analyzed by Quantitative Insights into Microbial Ecology, QIIME version 1.9.1 (available at http://qiime.sourceforge.net/; Caporaso et al., 2010). General pipelines for data analysis was previously described in details (Mandal et al., 2016). Forward and reverse ends sequences were joined together by using join_paired_ends.py command followed by formatting barcodes using customized Perl script, before extracting barcodes using extract_barcodes.py option. Demultiplexing and quality filtering were performed by split_libraries_fastq.py with default options. OTU picking was performed by using reference sequences from NCBI RefSeq 16S RNA database (O’Leary et al., 2016) and Swarm algorithm (Mahé et al., 2014). Taxonomic classification was performed by using reference taxonomy file from NCBI RefSeq 16S RNA sequences and SortMeRNA algorithm (Kopylova et al., 2012). NCBI RefSeq 16S RNA sequences are curated, non-redundant and quality controlled (Pruitt et al., 2007; O’Leary et al., 2016). We used this database instead of greengenes database for better taxonomic assignment at species level. Cumulative sum scaling (CSS) method with QIIME was used to normalize the OTU BIOM (biological observation matrix) before taxonomic assignment and alpha diversity calculation. Beta diversity estimates were calculated by using beta_diversity_through_plots.py options of QIIME with even sampling depth of 8000. Analysis of similarities (ANOSIM) between groups were performed using unweighted UniFrac distance metric (compare_categories.py, QIIME). Statistical significance in alpha diversity indices and different taxa among various groups were measured using one-way analysis of variance (ANOVA) followed by *post hoc* Student’s *t*-test using JMP Genomics 7 software.

## Results

After demultiplexing and quality filtering, there was 1,350,414 assembled sequence reads ranging from 444 to 574 bp with median sequence length 546 bp. Summarizing raw vs. CSS normalized otu biom table resulted in mean sample depth of 33,760.35 ± 3,311.22 and 1,488 ± 11.72 reads per sample, respectively. CSS normalized otu biom table was used further for taxonomy assignment and alpha diversity analysis.

### Taxonomy Assignment

#### Phylum Level

**FIGURE 1:**
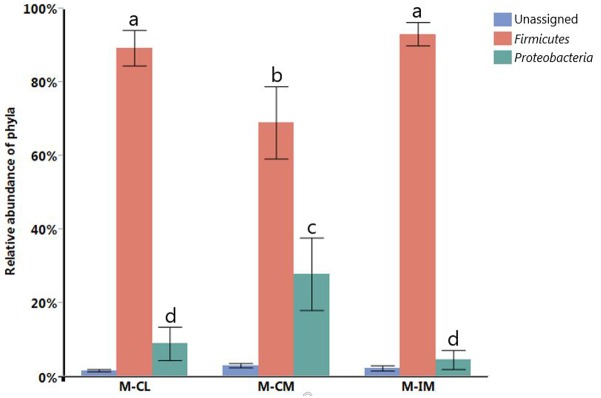
Relative abundance of different phyla. Different letters indicate significance at *p* < 0.05. Total bacterial cells from cecal lumen (T-CL). MRS-recovered cells from cecal lumen (M-CL), cecal mucosa (M-CM), and ileal mucosa (M-IM).

Taxonomic analysis among MRS groups revealed *Firmicutes* (83.83%) as the predominant phylum followed by *Proteobacteria* (13.83%). *Firmicutes* were found significantly higher in cecal lumen (M-CL) and ileal mucosa (M-IM) as compared to cecal mucosa (M-CM) at *p* < 0.05 (**Figure 1**), but there was no significant difference between M-CL and M-IM. On the contrary, *Proteobacteria* were found significantly higher in M-CM as compared to M-IM and M-CL at *p* < 0.05 (**Figure 1**).

#### Genus Level

**FIGURE 2:**
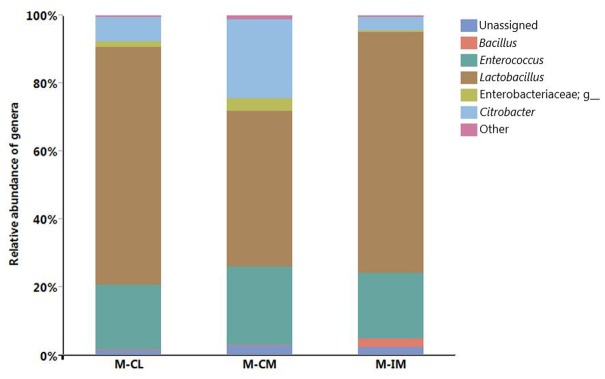
Relative abundance of different genera. MRS-recovered cells from cecal lumen (M-CL), cecal mucosa (M-CM), and ileal mucosa (M-IM).

Relative abundance of different genera recovered from MRS groups (≥1% of all MRS groups) is shown in **Figure 2**. *Lactobacillus*, *Enterococcus*, and *Citrobacter* were the major predominant genera recovered from MRS groups. *Lactobacillus* was observed significantly higher in M-IM and M-CL as compared to M-CM (*p* < 0.01), whereas *Citrobacter* was significantly higher in M-CM as compared to M-IM (*p* < 0.05). Although *Lactobacillus* was predominant genus in each MRS group, recovery of other genera demonstrated that MRS agar medium also supports the growth of the strains belonging to *Enterococcus* and *Citrobacter.*

#### Species Level

**FIGURE 3:**
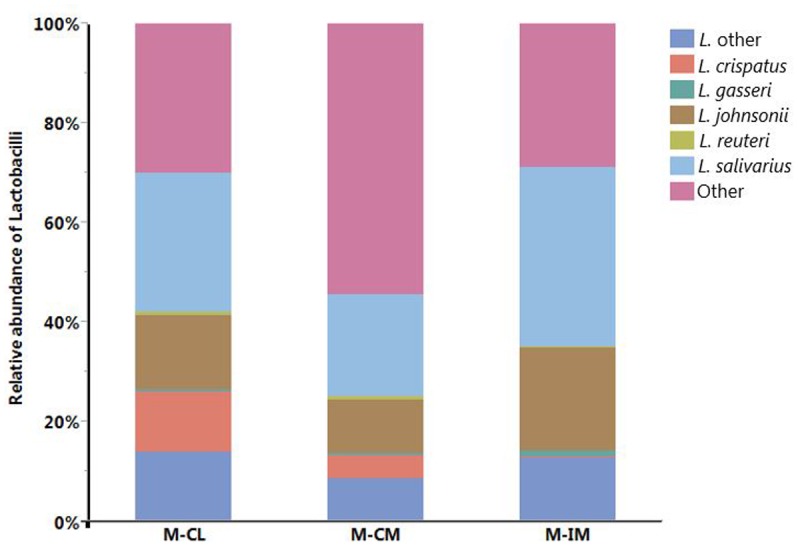
Relative abundance of different *Lactobacillus* species. MRS-recovered cells from cecal lumen (M-CL), cecal mucosa (M-CM), and ileal mucosa (M-IM).

Among the major *Lactobacillus* species identified, relative abundance of *L. salivarius* was highest in all three groups followed by *L. johnsonii*. Both *L. salivarius* (36%) and *L. johnsonii* (21%) were higher in M-IM as compared to M-CL (*L. salivarius*, 28%; *L. johnsonii*, 15%) and M-CM (*L. salivarius*, 20%; *L. johnsonii*, 11%) as shown in **Figure 3**. *L. crispatus* was found higher in M-CL as compared to M-CM and M-IM, but significant difference was found only between M-CL and M-IM (*p* < 0.01). Similarly, *L. gasseri* was found significantly higher in M-IM as compared to M-CM (*p* < 0.05).

### OTU Heatmap at Species Level

**FIGURE 4:**
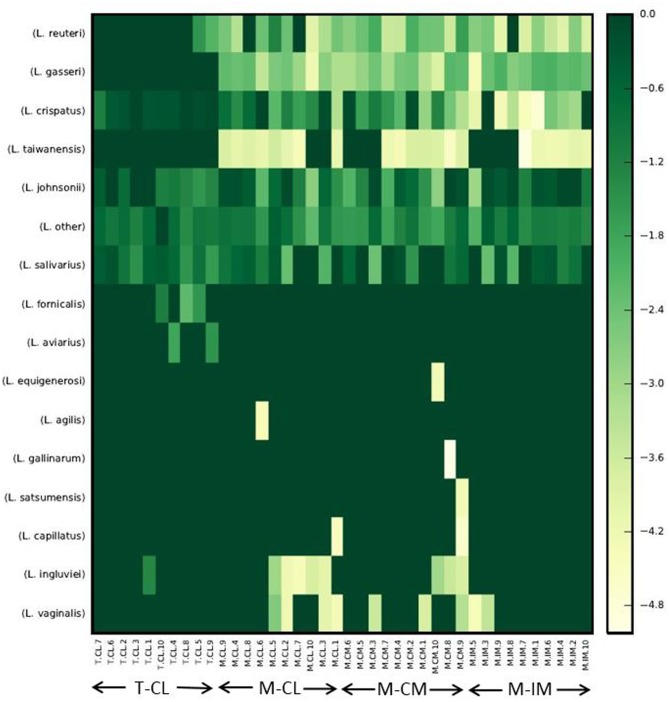
Heatmap of normalized OTU table consisting of *Lactobacillus* species only. Heatmap was constructed with make_otu_heatmap.py option of QIIME with log transformation where all zeros were set to a small value (1/2 the smallest non-zero entry), and data was translated to non-negative after log transformation, and num_otu_hits was set to 0. The abundance of *Lactobacillus* species decreases as the intensity of color decreases from green to yellow. Total bacterial cells from cecal lumen (T-CL). MRS-recovered cells from cecal lumen (M-CL), cecal mucosa (M-CM), and ileal mucosa (M-IM).

The OTU heatmap that consists of only *Lactobacillus* species, constructed with QIIME, revealed that *L. aviarius* and *L. fornicalis* were detected only from the total bacterial group (T-CL) as shown in **Figure 4**. Although these species were found only in a subset of T-CL samples, their relative abundance was significantly high as indicated by the green colors. Some other *Lactobacillus* species such as *L. aviarius*, *L. equigenerosi*, *L. agilis*, *L. gallinarum*, *L. satsumensis*, and *L. capillatus* were also detected in negligible amounts, in only one or two samples of the total bacterial group or MRS groups, which may be due to the errors during PCR or Illumina sequencing step.

#### Alpha Diversity

**FIGURE 5:**
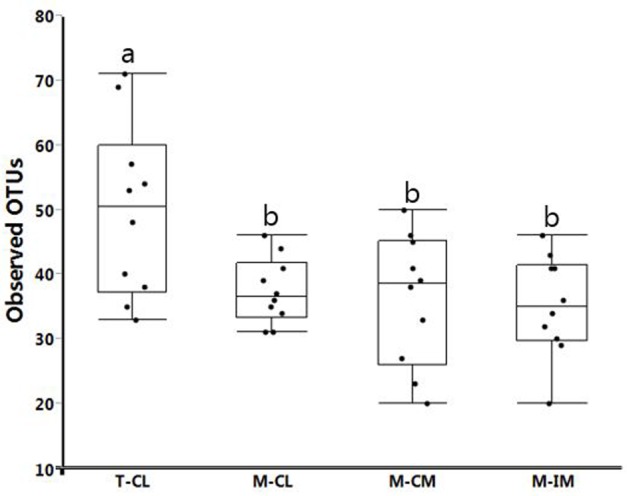
Alpha diversity in different groups measured with Observed_otus metric. Bars with different letters represent statistical significance at *p* < 0.01. Total bacterial cells from cecal lumen (T-CL). MRS-recovered cells from cecal lumen (M-CL), cecal mucosa (M-CM), and ileal mucosa (M-IM).

The observed OTUs ranged from 20 to 71 for all samples together. The alpha diversity measured with observed OTU metric was not significantly different among M-CL, M-CM, and M-IM. But as expected, the alpha diversity was significantly higher in the samples for which genomic DNA was directly isolated from total bacteria (T-CL) as compared to the samples recovered from MRS medium at *p* < 0.01 as shown in **Figure 5**.

#### Beta Diversity

**FIGURE 6:**
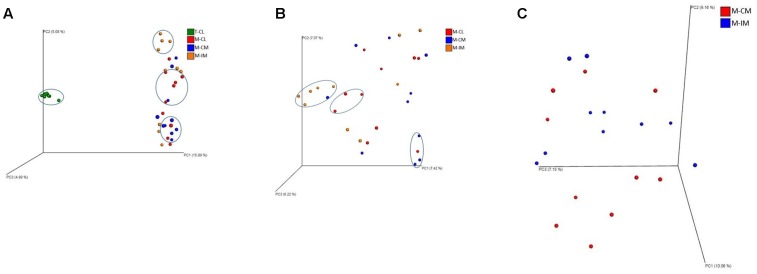
PCoA plots showing significant difference in bacterial community structure. **(A)** Among all groups analyzed; MRS-recovered cells from cecal lumen (M-CL), cecal mucosa (M-CM) and ileal mucosa (M-IM), and total bacterial cells from cecal lumen (T-CL) (*R* = 0.67, *p* = 0.001). **(B)** Among MRS groups; M-CL, M-CM, and M-IM (*R* = 0.13, *p* = 0.01). **(C)** Between two different regions of gut; M-CM and M-IM (*R* = 0.18, *p* = 0.02).

Unweighted unifrac distance metric was used to calculate ANOSIM. ANOSIM results showed that there were significant differences in bacterial community structure among different groups (M-CL, M-CM, M-IM, and T-CL; *R* = 0.67, *p* = 0.001) as illustrated in PCoA plot in **Figure 6A**. Similarly, the difference in bacterial community structure was observed among the groups of samples isolated from MRS medium (M-CL, M-CM, and M-IM; *R* = 0.13, *p* = 0.01) as shown in **Figure 6B**, and also between cecal and ileum mucosal samples (*R* = 0.18, *p* = 0.02) as shown in **Figure 6C**.

## Discussion

Although the use of different species of *Lactobacillus* as probiotics in chickens has shown beneficial effects (Zhang et al., 2007; Mappley et al., 2013; Saint-Cyr et al., 2017), there is still a lack of solid scientific basis for probiotic actions, and thus effective strategies to isolate promising probiotic strains. Comprehensive investigation of *Lactobacillus* populations in chicken GIT might provide important insights for better understanding of their roles in host function, and therefore for development of better screening strategies to identify more effective probiotic strains. Comprehensive characterization of chicken gut microbiota through the use of high throughput next generation sequencing (HT-NGS) has been limited as compared to human gut microbiota (Shaufi et al., 2015). It has already been reported that the relative abundance of *Lactobacillus* varies among different segments of the GIT in chickens (Gong et al., 2007; Ranjitkar et al., 2016) using culture independent method. Only one study reported the analysis of mucosa associated microbiota in chicken GIT via high-throughput sequencing of 16S rRNA gene sequences (Gong et al., 2007). Thus, there is very limited information available regarding topological differences of *Lactobacillus* population found in chicken GIT.

Gong et al. (2002) reported differences in bacterial populations between lumen and mucosa of chicken caeca through terminal restriction fragment length polymorphism (T-RFLP). The 16S rRNA gene-based analysis of mucosa-associated bacterial populations in chicken GIT revealed *Lactobacillus* as a predominant genera in upper GIT where *L. salivarius* and *L. aviarius* were predominant species in genus *Lactobacillus* (Gong et al., 2007). Similarly previous studies reported *Lactobacillus* species higher in ileum than cecum (Ranjitkar et al., 2016; Wang et al., 2016). We also noticed higher percentage of *L. salivarius* and *L. johnsonii* in ileal mucosa as compared to cecal lumen and cecal mucosa, albeit there was no significant differences among them. This is in agreement with our findings at phylum level where *Firmicutes* were higher in ileal mucosa as compared to cecal lumen and cecal mucosa, but significant difference was observed only between ileal mucosa and cecal mucosa. Observation of other genera that do not belong to lactic acid bacteria (LAB), such as *Citrobacter* and *Bacillus*, among the MRS groups suggests the limited selectivity of MRS agar for LAB strains as demonstrated earlier (Hartemink and Rombouts, 1999; Quartieri et al., 2016). Our report on the limited selectivity of MRS agar should be considered carefully when MRS agar is used as a means to estimate CFUs of LAB strains present in animal GIT samples. We reported *L. salivarius* to be a predominant species in all regions and locations of the GIT, which is in agreement with recent studies in chickens that reported higher percentage of *L. salivarius* in both cecum and ileum at the age of 36 (Ranjitkar et al., 2016), and at ileal mucosa at the age of 35 (Wang et al., 2016). These recent findings are in agreement with the previous reports that *L. salivarius* are consistently detected in older birds (Knarreborg et al., 2002; Guan et al., 2003). In this study, *L. crispatus* was found significantly higher in cecal lumen than ileal mucosa whereas *L. gasseri* was found significantly higher in ileal mucosa as compared to cecal mucosa. *L. crispatus* can be found in vertebrate GIT and is a *Lactobacillus* species frequently isolated from human vaginal tract (Witkin et al., 2007; El Aila et al., 2009). However, we should consider different factors including age, diet, litter type, horizontal gene transfer, chicken type, geography, climate, environment, feed additive, etc. before direct comparison of the present study with other findings, since these factors can affect chicken GIT microbiota (Qu et al., 2008; Danzeisen et al., 2011; Wang et al., 2016).

We observed *L. aviarius* and *L. fornicalis* only in total bacterial group. Failure to recover these species from MRS agar may be due to the followings reasons; these species either (i) require strictly anaerobic condition (*L. aviarius*), or (ii) grow well under anaerobic condition although being facultative anaerobic (*L. fornicalis*) as compared to microaerophilic condition at 37°C, which was used in this study (Fujisawa et al., 1984; Dicks et al., 2000; Baele et al., 2003). Alternatively, some of these species may have unique metabolic requirements that are not provided in MRS media. Observation of significantly higher alpha and beta diversity in total bacterial group (T-CL) as compared to MRS groups is obvious. Among the MRS groups (M-CL, M-CM, and M-IM), alpha diversity was observed higher in cecal lumen followed by cecal mucosa and ileal mucosa, although there was no significant difference. This was in agreement with ANOSIM results which showed differences in bacterial community structure among different MRS groups. Thus results from both alpha and beta diversity revealed difference in bacterial diversity between cecum and ileum, which is similar with the previous findings (Shaufi et al., 2015; Ranjitkar et al., 2016).

In summary, *L. salivarius* was found as a dominant species in all three regions of the GIT. Relative abundance of *Lactobacillus* not only varied with different regions of the GIT but also varied between lumen and mucosa of the same region. All the *Lactobacillus* species present in chicken GIT samples may not be cultured on MRS agar media. Analysis of alpha diversity and beta diversity revealed differences in the structure of MRS-recovered bacterial communities among different regions and locations of the GIT.

To our knowledge, in most studies to isolate effective probiotics in poultry as well as in other food-producing animals, the first step is isolation of strains that belong to the target taxonomic group (e.g., *Lactobacillus* genus), followed by a screening of the strains for various desirable phenotypes, including resistance to acidic pH or bile acid, ability to inhibit the growth of pathogenic bacteria *in vitro*, and particular enzymatic properties among others (Asghar et al., 2016; Kizerwetter-Świda and Binek, 2016). However, this approach has the following inherent limitations: (1) the screening is conducted with randomly picked strains from a large pool of bacterial strains, (2) the number of strains screened is critically limited due to the labor and time required for the process, and (3) the suitability of the screening criteria for *in vivo* efficacy remains questionable (Morelli, 2000). For these reasons, this current approach remains ineffective, limiting our ability to exploit the gut microbiota as a rich resource for development of more effective probiotics.

On the other hand, the use of culture-independent approaches (16S rRNA gene profiling, and shot-gun metagenome analysis) have provided new insights on the function of gut microbiota in overall body functions (Singh et al., 2014; Choi et al., 2015; Yan et al., 2017), and are expected to reveal some core members of gut microbiota that play crucial roles in promoting gut health and thus growth performance in poultry. For example, Stanley et al. (2016) attempted to identify probiotic candidates for broilers based on their association with desirable productivity outcomes using microbiota analyses. Although the lack of consistency in the microbial shifts across the three animal trials was shown as a major challenge for this effort, this new approach demonstrated in Stanley et al. (2016) has a great potential for identification of effective probiotics. On the other hand, Buffie et al. (2015) identified *Clostridium scindens* as a species associated with resistance to *C. difficile* gut colonization in both mice and humans using comparative microbiota analysis and mathematical modeling, and experimentally demonstrated that oral administration of *C. scindens* significantly enhanced resistance to *C. difficile* colonization in mice.

In the study by Buffie et al. (2015) the use of the *C. scindens* strain originated from different source was successful in demonstrating the probiotic efficacy, suggesting that the genetic capacity conferring resistance is probably well-conserved within the *C. scindens* species. However, an increasing body of studies are pointing to the fact that intra-species variations on genetic capacity is quite common (Greenblum et al., 2015). In some cases, different strains from the same species can act in an opposite manner as previously reported by Fåk and Bäckhed (2012) that *L. reuteri* ATCC PTA 4659 was linked to weight loss while *L. reuteri* L6798 was linked to weight gain in mice. These findings suggests that the probiotic candidates identified by comparative microbiota analysis should be strain-specific in some cases and thus need to be isolated from appropriate samples used for the microbiota analysis.

However, when the target species or strains are identified, the next step to isolate the strains represented by the identified signature DNA sequences (e.g., specific 16S rRNA gene sequences) would encounter multiple challenges to overcome, primarily due to the complex microbiota background from which the target strains are to be isolated. One major challenge can be the culturability of the target strains, because DNA sequence data do not provide information regarding culturability of each member of a microbiota. However, a comparative microbiota analysis between culture-recovered bacteria such as shown in our study (e.g., M-CL) and direct microbiota (e.g., T-CL) can identify the culturable members in the microbiota as illustrated in **Figure 4**. This information would ensure that the efforts to retrieve target strains is an achievable goal, although the practical strategies to isolate the strains based on DNA signatures still remains to be developed.

We reason that the conventional approach to isolate probiotics should move toward this new direction to fully exploit gut microbiota in poultry as a valuable resource to develop probiotics that would be more effective in positively modulating gut microbiota, thereby preventing diseases, and promoting health and growth performance in poultry. Our study is conducted on a small scale, but it is the first attempt to define MRS-recovered *Lactobacillus* subpopulations in GIT of chickens with the long-term goal of developing more effective *Lactobacillus* probiotic candidates based on system-wide comparative microbiota analyses.

## Author Contributions

BA and YK designed the experiment. BA conducted the study, analyzed the data and wrote the manuscript. BA and YK revised the manuscript.

## Conflict of Interest Statement

The authors declare that the research was conducted in the absence of any commercial or financial relationships that could be construed as a potential conflict of interest.
